# Viability of *Lactobacillus plantarum* on Fresh-Cut Chitosan and Alginate-Coated Apple and Melon Pieces

**DOI:** 10.3389/fmicb.2018.02538

**Published:** 2018-10-23

**Authors:** Barbara Speranza, Daniela Campaniello, Antonio Bevilacqua, Clelia Altieri, Milena Sinigaglia, Maria Rosaria Corbo

**Affiliations:** Department of the Science of Agriculture, Food and Environment, University of Foggia, Foggia, Italy

**Keywords:** probiotic, fresh-cut, edible coating, shelf life, fruit pieces

## Abstract

There is an increasing trend toward foods with probiotics; the awareness of healthy diet and wellbeing is the leading cause of this increase. As a result, food producers and stakeholders require new probiotic products. The increased incidence of lactose intolerance and the new lifestyles (vegan and vegetarian styles) have led to a renewed interest in non-dairy probiotic carriers. The use of biopolymeric matrices to develop active food packaging carrying probiotics has been studied and proposed as an alternative method to design new solutions.

The main topic of this paper was the design of fresh-cut fruits (apples and melons) as carriers for a promising *Lactobacillus plantarum*; fruit pieces were coated with either alginate or chitosan. Apple (Granny Smith) and melon pieces (*Cucumis melo*, var. *Cantalupensis*) were preliminary treated with an anti-browning solution (citric and ascorbic acids). Then, fruit pieces were dipped in a solution containing *L. plantarum* c19 (9 log cfu/ml) and coated with alginate or chitosan. Samples without probiotic and/or coatings were used as controls.

All samples were stored at 4°C for 14 days under air or modified atmosphere (65% N_2_, 30% CO_2_, and 5% O_2_); the following analyses were done: pH, color, O_2,_ and CO_2_ in the head space, microbiology (mesophilic bacteria, lactic acid bacteria, yeasts, and molds).

The most important results can be summarized as follows: (a) Alginate coating showed better performances than chitosan-coating, as it did not affect the viability of *L. plantarum*. (b) The inoculation of probiotics in the controls negatively affected the color, but the coating was able to counteract this effect. This paper supports the combination of edible coatings and probiotic as a promising way to design new fruit-based functional foods; further investigations are required to study the effect of this combination on the sensory scores.

## Introduction

A probiotic is “a live microorganism that, when administered in adequate amounts, confers a health benefit on the host” (FAO/WHO, 2001). Many microbes and applications fit to this definition, as they possess the three main requisites of a probiotic: microbial status, viability, and benefits to host. Lactobacilli represent a significant part of human microbiota; moreover, they possess/exert some important effects: antimicrobial activity, enhancement of immunity, and antitumorigenic activities. The most important lactobacilli of gut are *Lactobacillus acidophilus*, *L. salivarius*, *L. casei*, *L. plantarum*, *L. fermentum*, and *L. brevis. L. plantarum* is also the predominant species in many fermented foods, both of animal, and vegetal origin; in particular, the species *L. plantarum* was recovered in fermented Italian green olives (Bevilacqua et al., 2010). Bevilacqua et al. (2010) isolated from Italian table olives “Bella di Cerignola” *L. plantarum* c19, a strain with some promising functional traits (growth at pH 4.0–9.0, survival in presence of 10% NaCl, antimicrobial activity against *Escherichia coli* O157:H7, adhesion to IPEC-J2 cells line and survival during the simulation of the transit into the gut) (Bevilacqua et al., 2010; Altieri et al., 2011). Nevertheless, this strain showed technological robustness and a prolonged viability in vegetable matrices (Perricone et al., 2010) and a strong antimicrobial activity toward some yeasts, usually found on vegetables (Bevilacqua et al., 2013). Thus, it was chosen as a model “functional” microorganism to design a new kind of probiotic carriers.

Probiotics are usually carried by means of fermented milks, dairies, or pharmaceuticals. However, the increased incidence of lactose intolerance, allergies to milk’ proteins, some concerns on cholesterol and the worldwide spreading of new life-styles (vegans and vegetarians) are the leading causes for an increased interest toward non-dairy probiotic foods, like the vegetal vehicles for probiotics (table olives, salted gherkins, and sauerkraut) (Granato et al., 2010; Vijaya Kumar et al., 2015).

The term “fresh cut” usually means vegetables ready to use (washed, cut, and fresh packaged). The International Fresh-cut Produce Association (IFPA) defines as “fresh cut” a fruit or vegetable, cut, and packaged, 100% eatable, with high nutritional value, ever ready and similar in taste to the fresh one (Lamikanra, 2002).

Fresh cut fruits show more problems than the whole ones; in fact, whole fruits are protected from microbial contamination for the presence of wax, peel, and others protective parts. Microorganisms are located on the surface and comprise bacteria (*Pseudomonas*, *Erwinia*, *Enterobacter*, *Lactobacillus* spp., etc.), molds (*Rhizopus*, *Aspergillus*, *Penicillum*, *Eurotium*, and *Wallemia*), yeasts (*Saccharomyces*, *Zygosaccharomyces*, *Hanseniaspora*, and *Candida*), viruses and parasites (Kalia and Gupta, 2006). Nevertheless, several intrinsic and extrinsic factors, may affect microbial population on fruit, influencing shelf life and/or allowing pathogen growth; for example, many *Salmonella* and *E. coli* O157:H7 infections are documented (Food and Drug Administration [FDA], 2010). Thus, fresh cut fruits have a lower shelf life, because of operations damaging tissues (peeling, cutting, and stoning), enhancing browning, softening, and spoilage (Chien et al., 2007).

Many authors proposed fruit pieces (apple, melon, pear, cashew, papaya, passion fruit, and fruit salads) as carriers for lactic acid bacteria and yeasts for either food fermentation or probiotic delivery with a focus on the viability of the starter/probiotic cultures, and on the sensory attributes (Kourkoutas et al., 2005; Tsakiris et al., 2006; Corbo et al., 2013; Martins et al., 2013; Gallo et al., 2014; Santos et al., 2017; Nikolau et al., 2019).

The market request is for a lower and lower presence of chemical preservatives in food, and food industry is responding by using more and more natural preservatives, able to control microbial growth. Several natural compounds have been used on fresh cut fruits, as well as phenols, chitosan, organic acids, and aldeids (Lanciotti et al., 2004), essential oils (González-Aguilar et al., 2010), applied by coating or by dipping (Lanciotti et al., 2004; Allende et al., 2006; Chien et al., 2007; Rico et al., 2007; Campaniello et al., 2008; González-Aguilar et al., 2010; D’Amato et al., 2010), and also using modified atmospheres (MAP), physical treatments, as well as high temperatures, irradiations, etc.

A promising way to improve the nutritional and the sensory traits of fruit and fruit pieces is the use of edible coatings, based on different polymers and materials (among others alginate, pectins, gellan, chitosan, caseinate, and k-carrageenate) (Campaniello et al., 2008; Moreira et al., 2015; López Aguayo et al., 2016; Sendurk Parreidt et al., 2018). However, to the best of our knowledge few data are available on the combination probiotics/edible coatings on fruit pieces and on the use of coatings as a way to overcome some drawbacks in the inoculation of probiotics in fruits.

Therefore, the aim of this work is to study and suggest a non-dairy probiotic carrier, combining the use of either apple or melon and an edible coating (chitosan or alginate), by focusing on the viability of probiotics, as a function of the coating and of the packaging atmosphere, as well as on the suitability of coatings to delay the changes on color throughout time.

## Materials and Methods

### Strain

*Lactobacillus plantarum* c19 (Bevilacqua et al., 2010) was at -20°C in MRS broth (Oxoid, Milan, Italy) +33% of sterile glycerol (J.T. Baker, Milan, Italy). Before each experiment, the strain was cultured in MRS broth (37°C for 24 h under anaerobic conditions).

### Sample Preparation

Apples var. Granny Smith were washed by hand under drinkable water (containing 0.2 mg/l free clorine, according to Italian law), peeled and cut (ca. 2 cm × 3 cm). Melons (sp. *Cucumis melo*, var. *Cantalupensis*) were peeled, sliced, and cut (ca. 2 cm × 3 cm).

Dipping solutions for the different treatments are reported in Table 1; the treatments are in Table 2. All samples were dipped in the AB solution (anti-browning solution) (15 min), except for chitosan-coated samples, because the anti-browning compounds were dissolved into the chitosan solution. After the anti-browning treatment, the samples were treated with the solutions reported in Table 2 and air-dried for 15 min. Chitosan coating was used only for apple pieces.

**Table 1:** Dipping solutions.

Dipping solutions	Contents
AB	Citrate (0,2% w/v), ascorbate (1% w/v), sterile distilled water
(anti-browning for every kind of sample)	
P (9 log cfu/ml probiotic)	*Lactobacillus plantarum* culture was centrifuged (3000 g for 15 min at 4°C) (centrifuge ALC 4239R, ALC, Milan, Italy). Cells were harvested and resuspended into sterile distilled water
A (alginate coating)	Alginate powder (2% w/v) melted into sterile distilled water at 80°C
CaCl_2_ (hardening)	CaCl_2_ (0.5% w/v)
CH (chitosan)	chitosan (1% w/v) melted into acid solution AB
AP	A + P
CHP	CH + P


**Table 2:** Sample preparation. The ratio fruit pieces/solution was 5 pieces in 200 ml of dipping solution.

	AB	P	A	AP	CaCl_2_	CH	CHP	Packaging in air	Packaging in mod. atm.
O	Yes	–	–	–	–	–	–	Yes	–
PO	Yes	Yes	–	–	–	–	–	Yes	–
AO	Yes	–	Yes	–	Yes	–	–	Yes	–
APO	Yes	–	–	Yes	Yes	–	–	Yes	–
CHO	–	–	–	–	–	Yes	–	Yes	–
CHPO	–	–	–	–	–	–	Yes	Yes	–
M	Yes	–	–	–	–	–	–	–	Yes
PM	Yes	Yes	–	–	–	–	–	–	Yes
AM	Yes	–	Yes	–	Yes	–	–	–	Yes
APM	Yes	–	–	Yes	Yes	–	–	–	Yes
CHM	–	–	–	–	Yes	–	Yes	–	Yes
CHPM	–	–	–	–	–	Yes	Yes	–	Yes


### Sample Packaging

After the preparation, the samples were packed in high-barrier plastic bags [nylon/polyethylene, 102 μm (Tecnovac, San Paolo D’Argon, Bergamo, Italy)]. The packaging was done through a S100-Tecnovac equipment. The technical characteristics of the bags were as follows: 170 mm × 250 mm; CO_2_ and O_2_ permeability of respectively, 3.26 × 10^-19^ and 9.23 × 10^-19^ mol m m^-2^ s^-1^ Pa^-1^ and water vapor transmission rate of 1.62 × 10^-10^ kg m^-2^ s^-1^. The samples were packaged in air (O) or in MAP (M: 65% N_2_, 30% CO_2_, and 5% O_2_).

The samples were stored at 4°C for 14 days and microbiological, and physico-chemical analyses were performed. All analyses were performed twice on two independent samples.

### Microbiological Analyses

The first homogenate was prepared with 25 g of fruits and 225 ml sterile saline solution (0.9% NaCl) in a Stomacher bag (Seward, London, United Kingdom) through a Stomacher Lab Blender 400 (Seward). The following analyses were done: (a) total bacterial count on Plate Count Agar (PCA, Oxoid, Milan, Italy) (32°C for 48 h); (b) psychrotrophic bacteria (PCA, 5°C for 1 week); (c) lactic acid bacteria MRS Agar 0.17 g/l of cycloheximide (Sigma-Aldrich, Milan) (37°C for 4 days, anaerobic conditions); (d) yeasts and molds on Sabouraud Dextrose Agar (Oxoid, Milan, Italy) incubated at 28°C for 2–5 days, respectively.

Five to ten colonies were randomly selected from MRS Agar from the samples inoculated with *L. plantarum* c19; DNA was extracted and PCR was run as reported by Bevilacqua et al. (2010).

### pH

Determination of pH was performed by a pH-meter Crison, model micro pH 2001 (Crison, Barcellona, Spain) on the homogenate prepared for the microbiological analyses.

### Colorimetric Analysis

Color was measured using a tristimulus colorimeter CR-300 Minolta Chromameter-2 Reflectance (Minolta, Japan). Data were expressed according to CIELAB scale: L (luminescence), a (red/green coordinate), and b (yellow/blue coordinate). The calibration of colorimeter was done on standard white (*L*^∗^ = 97,03, *a*^∗^ = +0,01, and *b*^∗^ = +1,63).

### Head Space Analysis

Quantitative analysis of O_2_ e CO_2_ in head space was performed using a PBI Dansensor (Checkmate 9900, Ringsted, Denmark), with test volumes of 10 cm^3^.

### Statistic

The results were analyzed by a two-way ANOVA and Tukey’s test as the *post hoc* comparison test (*P* < 0.05).

## Results

LAB were below the detection limit on the uninoculated samples (controls: samples O, AO, CHO, M, AM, and CHM), while the identification of some isolates from inoculated samples (samples PO, APO, CHPO, PM, APM, and CHPM) confirmed that LAB population was composed by *L. plantarum* (data not shown).

**FIGURE 1:**
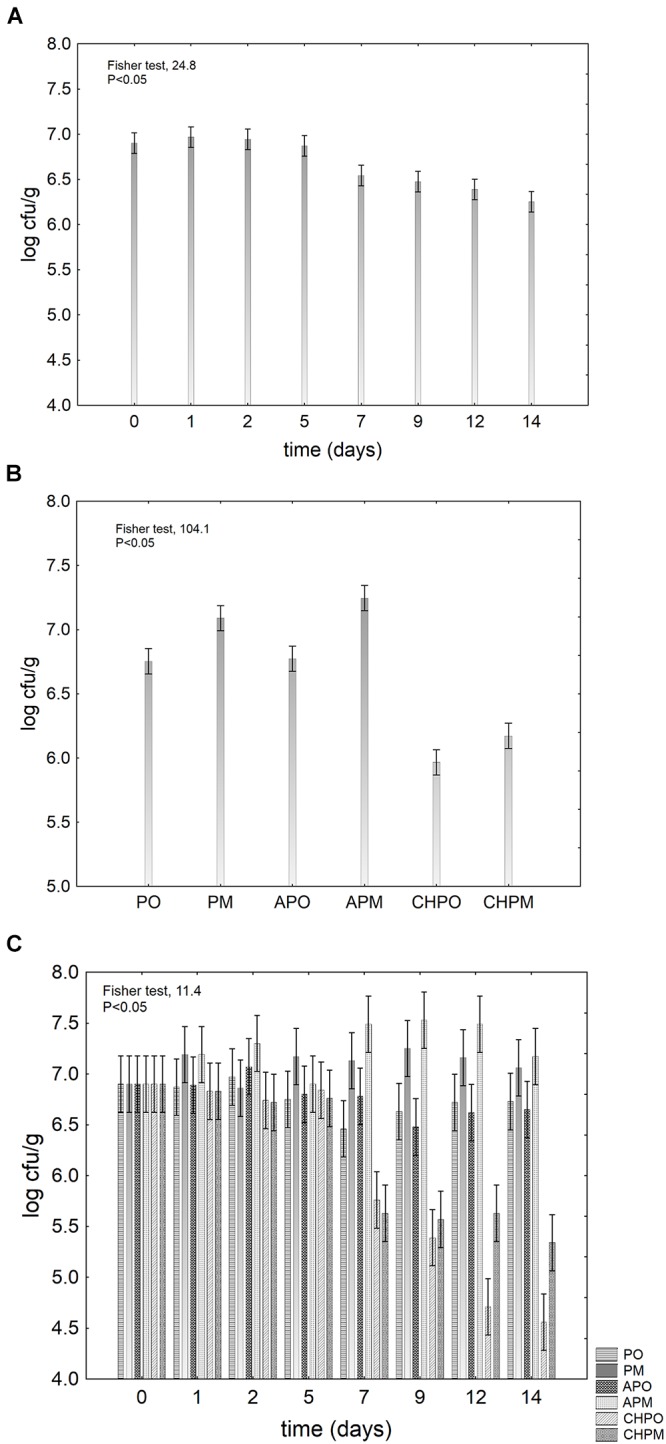
Decomposition of the statistical hypothesis for the effect of the kind of treatment and the storage time on the viability of *Lactobacillus plantarum* c19 on apple pieces. Vertical bars denote 95% confidence intervals. **(A)** effect of the storage time; **(B)** effect of the treatment; **(C)** interaction time/treatment. For the acronyms of samples see Materials and Methods.

The results for the viable count of *L. plantarum* c19 on apple pieces were analyzed by two-way ANOVA, using the treatment (kind of samples) and the time (duration of storage) as categorical predictors. The quantitative output of this statistic can be found in the graphs of the decomposition of the statistical hypothesis (Figure 1).

As expected, the storage time negatively acted on probiotic, as it caused a decrease of 1 log cfu/g (Figure 1A). In addition, the different treatments affected the viability of *L. plantarum* in a different way and the coating with chitosan, both in air and under MAP, caused a mean decrease of the target of 1 log cfu/g (Figure 1B). Both Figures 1A,B do not show actual values, but they only offer an insight on the quantitative effect of each factor of the design.

The effective trends of *L. plantarum* c19 are in Figure 1C; at the beginning, the viable count of the probiotic was 6.8 log cfu/g. Then, it experienced a strong decrease in chitosan coated-apple pieces up to 4.5 log cfu/g in air and 5.3 log cfu/g under MAP.

The legal break point for probiotics in food has been set to 10^7^ cfu/g or 10^9^ per day (Rosburg et al., 2010; Italian Ministry of Health, 2013). However, the main goal of this step was not to assess a “functional” shelf life (time to main the viability of the probiotic to an acceptable level), but to assess the suitability of coatings and to choose the best combination probiotic/coating. However, the low level of probiotic (<7 log cfu/g) suggests that the inoculation should be further improved to increase the concentration of *L. plantarum* on the inoculated samples.

Concerning the other microbiological data, moulds, yeasts, and psychrotrophic bacteria were below the detection limit for the whole storage time (data not shown).

**FIGURE 2:**
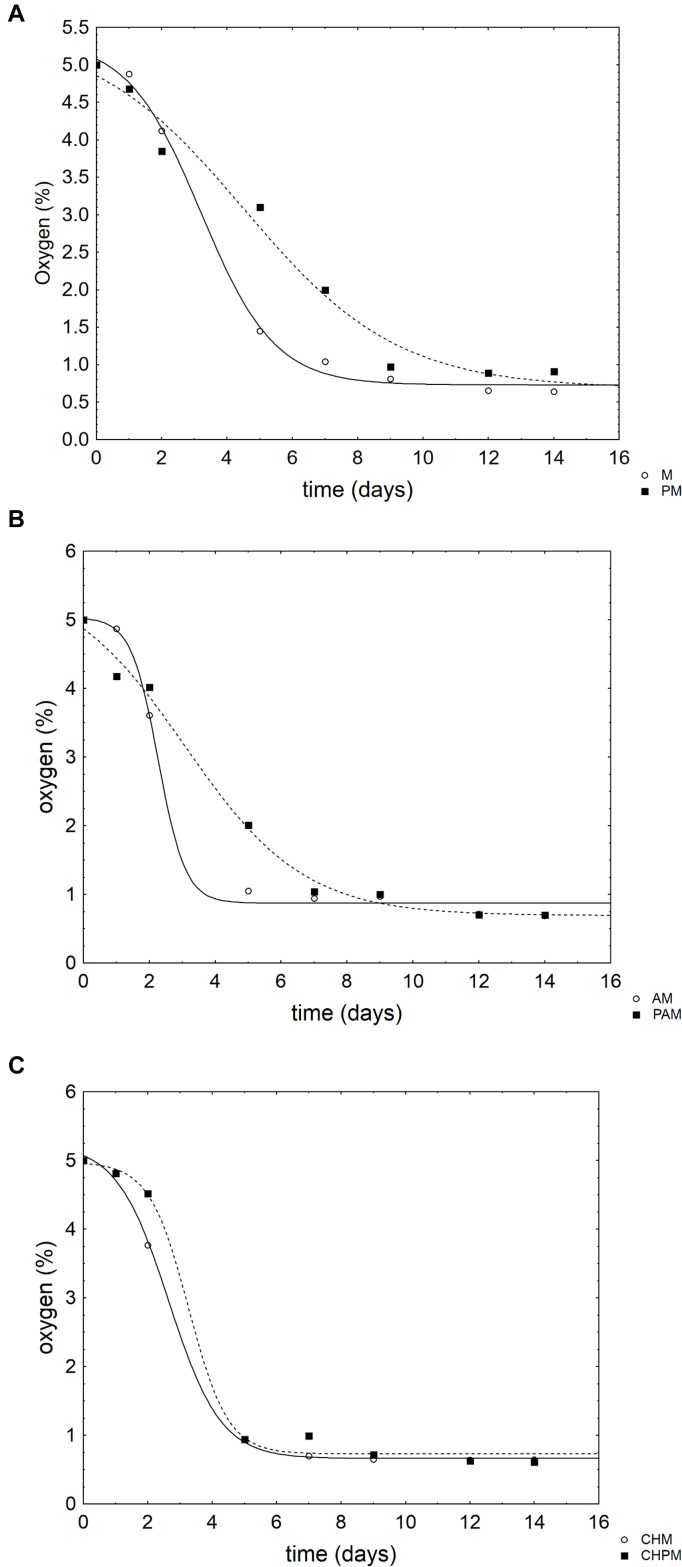
Evolution of oxygen (v/v) in the head space of the samples of apple pieces packed in the modified atmosphere. The points represent the mean of two replicates. The lines represent the best fit through a negative Gompertz equation. For the acronyms of samples see Materials and Methods. **(A)** Apple pieces; **(B)** Apple pieces with alginate; **(C)** Apple pieces with chitosan.

Apple pieces were also analyzed in relation to physico-chemical parameters. Concerning the gases in the head-space, the samples packed in MAP showed a CO_2_-trend like a negative sigmoid; therefore, the results were modeled with a negative Gompertz equation. In the controls (apple pieces without coating) (Figure 2A) and in those coated with alginate (Figure 2B), the probiotic exerted a significant effect on the rate of oxygen consumption and decreased it (from 0.88 to 0.42% O_2_/day in the controls and from 1.03 to 0.55% O_2_/day in alginate coated-apple pieces). This effect was not found in the chitosan coated samples (Figure 2C).

In the samples packed in MAP there was a linear decrease of O_2_ up to 7–10% without significant differences amongst the samples (data not shown). CO_2_ increased to 10–15% in the samples packed in air and to 35–36% for the samples packed in MAP (data not shown).

**FIGURE 3:**
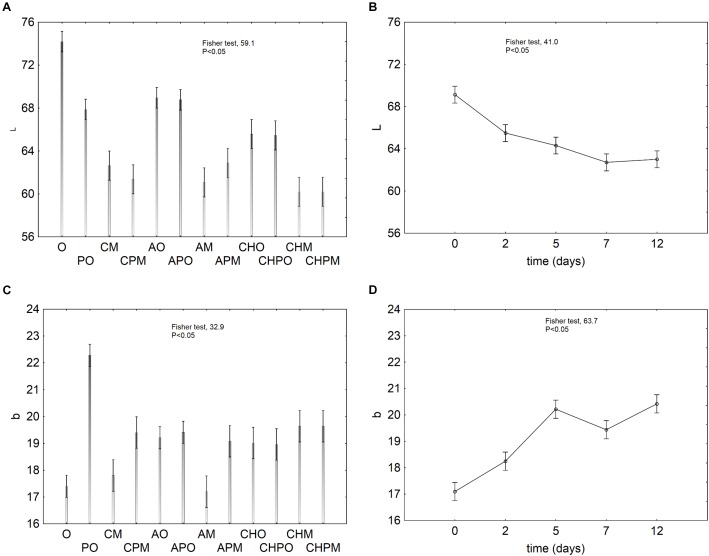
Decomposition of the statistical hypothesis for the effect of the kind of treatment and the storage time on the instrumental color. Vertical bars denote 95% confidence intervals. For the acronyms of samples see Materials and Methods. **(A)** effect of the treatment on L; **(B)** effect of the storage time on L; **(C)** effect of the treatment on b; **(D)** effect of the storage time on b.

The effect of the probiotic on color was analyzed by the evaluation of the decomposition of the statistical hypothesis on the parameters L (luminescence) and b (yellow/blue coordinate). The effect of the treatment on L was variable and relied upon the kind of treatment itself (Figure 3A). In the controls packed in air (O and PO), the inoculation of *L. plantarum* c19 caused a strong decrease of L; in the samples packed in MAP (M and PM) the decrease of L was independent from the inoculation of probiotic and was probably due to the atmosphere. A higher value of L was found in alginate-coated apple pieces packed in air. As expected, the storage time negatively acted on L because of enzymatic browning (Figure 3B).

Figures 3C,D show the effect of the treatment and storage time on the parameter b. The inoculation of *L. plantarum* caused an increase of b in the controls (see the difference between O and PO); on the other hand, the increase was less pronounced in the samples coated with alginate and chitosan (Figure 3C). As expected, b increased within the storage because of the enzymatic browning (Figure 3D).

A second phase of this research was aimed at assessing the effect of probiotic and coating on melon pieces. Chitosan was not used due to the strong effect on the viability of *L. plantarum*, as aforementioned.

**FIGURE 4:**
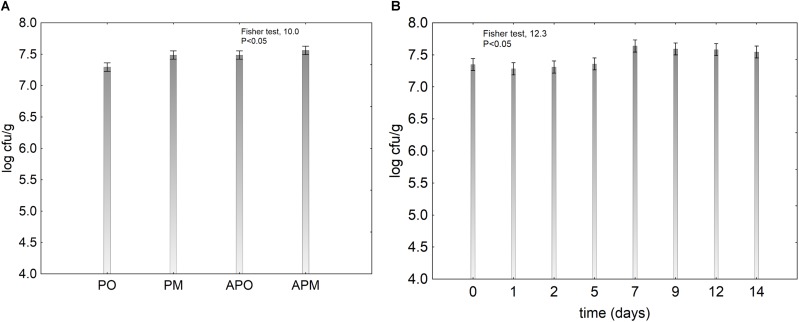
Decomposition of the statistical hypothesis for the effect of the kind of treatment and the storage time on the viability of *L. plantarum* c19 on melon pieces. Vertical bars denote 95% confidence intervals. For the acronyms of samples see Materials and Methods. **(A)** effect of the treatment; **(B)** effect of the storage time.

The effect of the treatment of the viability of probiotic was less significant (Figure 4A), whereas time exerted a significant effect as probiotic increased throughout the storage (Figure 4B). In the samples packaged in air and in MAP without coating, the pH decreased and the probiotic enhanced this effect: at the beginning the pH was 6.8 and after 14 days 5.0 in the controls and 4.5–4.3 in the samples with probiotics. The use of alginate-coating controlled this phenomenon and the final pH was 6.5 (data not shown).

**FIGURE 5:**
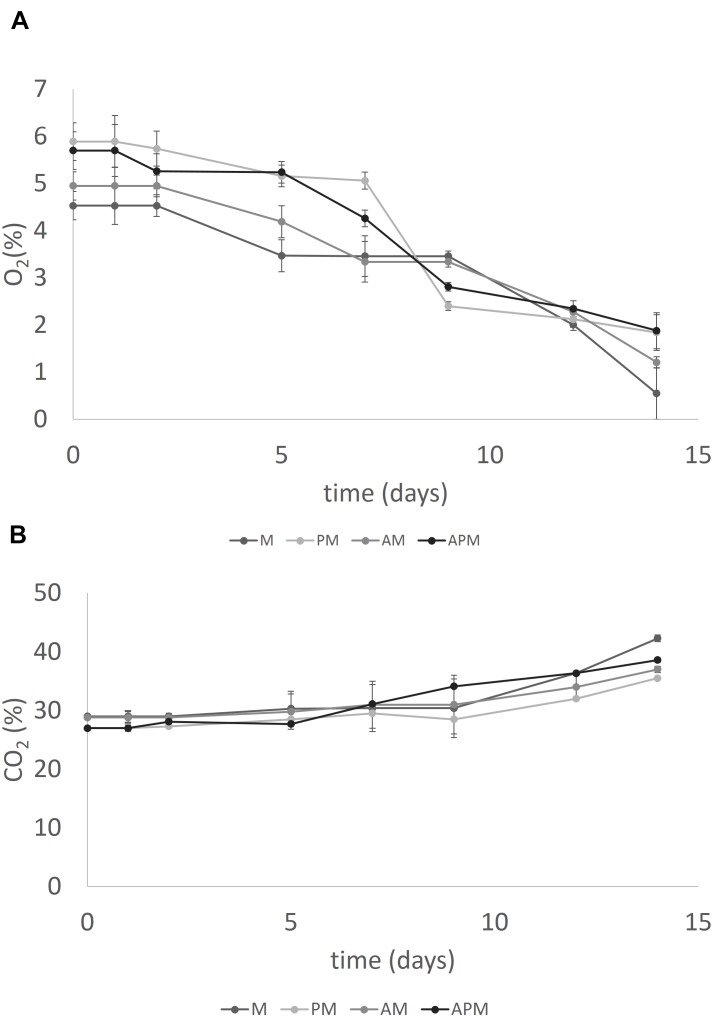
Evolution of O_2_ **(A)** and CO_2_ **(B)** in the head-space of melon pieces inoculated with *L. plantarum* c19 and packed in modified atmosphere. Mean ± standard deviation. For the acronyms of samples see Materials and Methods.

Oxygen levels decreased and carbon dioxide increased without significant differences amongst the samples. As an example, Figures 5A,B show the level of gasses in the samples packed in MAP.

## Discussion

The demand for new food-carriers for probiotics is only one of the reason of the emerging trend toward non-dairy food products, such as those based on fruits (Martins et al., 2013), with potential benefits for human health.

Consumers require more and more ready-to-eat and convenience foods and this is why their market increased in the last decade (Rojas-Graü et al., 2011). In this increasing trend, it is important to offer foods with the dual functionality of vegetable origin and probiotics.

The high preference of consumers for probiotic foods containing fruits has been reported by Espírito-Santo et al. (2011); moreover, fruits represent a good substrate for probiotics since they have nutrients (Soccol et al., 2010) and possess morphological structures favoring microbial growth (Martins et al., 2013). Another benefit is the lack of allergenic substances of dairy products.

The results obtained in the present study underlined that the presence of the tested probiotic strain on the apple pieces was well supported. However, the inoculation of probiotics in foods often requires special technologies; probiotics, in fact, retain an active metabolism and could cause an over-acidification of the product (Bevilacqua et al., 2016; Racioppo et al., 2017), with a worsening of the sensory trait. This effect was reported by Martins et al. (2015) on apple pieces containing *L. plantarum* and was confirmed in this research on apple pieces inoculated with the probiotic but without coating (for example the sample PO). Different strategies can be used to counteract this effect; hereby we propose the use of an edible coating, based either on alginate and chitosan and combined with MAP.

Nevertheless, the study evidenced that the kind of coating significantly affected the probiotic viability; in particular, chitosan reduced the viability of lactobacilli, as one could expect from the wide antifungal and antibacterial abilities (Altieri et al., 2005; Campaniello et al., 2008; Raafat and Sahl, 2009; Campaniello and Corbo, 2010).

On the other hand, alginate coating showed better performances by preserving the viability of *L. plantarum*; moreover, it was able to counteract the bad effect of the probiotic on apple pieces, both in air, and in MAP and achieved the primary goal of this research: to design a probiotic carrier and avoid the negative effects of lactobacilli.

The second phase of the study was focused on melon. First de Oliveira et al. (2014) proposed melon pieces as probiotic carriers and this research supports their suitability.

In this phase, the chitosan coating was not tested in the light of results obtained with apple samples. Alginate exerted a positive effect whatever the storage atmosphere and also counteracted the negative effect of lactobacilli on pH.

## Conclusion

The use of fruit-pieces (i.e., apple and melon), with a good combination of packaging atmosphere and coating can be considered a promising way to design new carriers for probiotic bacteria. In particular, alginate coating did not affect the viability of the model microorganism and to some extent could counteract the negative effect of probiotication on color.

Fresh-cut fruits are well accepted by consumers, indicating that are marketable products, having all the benefits provided by probiotic functional food, with the advantage that everybody can consume it. However, more studies and clinical trials are needed in order to evaluate id the probiotic carried out by vegetable foods exert their role in the gut.

Further investigations are also required to study the effect of probiotication on the sensory scores, as well as to increase the level of probiotics in order to fit to the legal requirements and drive the research from lab to market.

## Author Contributions

MC and MS conceived the study and funded the research. BS, DC, and CA designed the experiments. BS and DC performed the experiments. AB performed the statistic. CA and AB wrote the manuscript. All the authors interpreted the results and reviewed the paper.

## Conflict of Interest Statement

The authors declare that the research was conducted in the absence of any commercial or financial relationships that could be construed as a potential conflict of interest.
